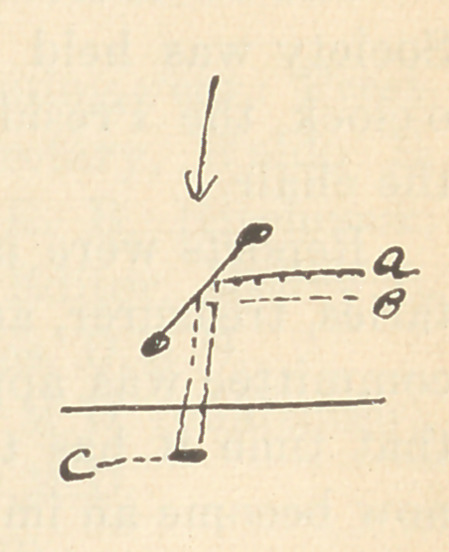# Reply to Review of Bödecker’s Book

**Published:** 1895-05

**Authors:** F. A. Roy


### Domestic Correspondence.

### REPLY TO REVIEW OF BÖDECKER'S BOOK.

To the Editor of the International Dental Journal:

Sir,—In your review of Bödecker's book, you charge that there is "no real evidence to prove the correctness of the theories advanced," and that the "views . . . are based solely on drawings in which the personal equation is a prominent factor, . . . for the drawings are all by Heitzmann." And you state that "drawing however beautifully done, and Heitzmann is a master in this direction, cannot carry conviction. A few photo-micrographs would have accomplished far more." Also, "it leads to the suspicion, whether justly founded or not, that either the slides cannot be represented or that they have no real existence." And, "it would seem that no expense should have been spared to meet this demand."

Heitzmann discovered the reticulum. He gives you drawings of it in the different tissues. This is the prime evidence. Others have seen and made drawings of the reticulum and speak and write of it. This is certainly good corroborative evidence. Then both here and in Europe are those who have seen and made drawings of the reticulum independently of Heitzmann. This is also corroborative: sections which show the reticulum have been photographed. A section must necessarily be examined at high powers of the microscope in order to see the reticulum. But no photograph yet produced of a section at such high powers can show what the eye can see. Even the thinnest section has a thickness which is very great when magnified by a high power, and the filaments of the reticulum do not lie on the surface nor all in one plane, but are in planes at innumerable angles with the line of sight. The reticulum is not an open network, but is embedded in a material not altogether transparent; so, for instance, take a filament at an angle of 45°, and at high powers only a very small portion is in focus, as at *a*, *b*, and would only show as a dot or very short line, *c*, unconnected with anything else, whereas the eye could follow that line by focussing, and the observer, if expert, could of course follow out such lines and draw what he sees, and in that way show the reticulum as it is seen.

We hope at an early day to succeed in making a photograph just as the drawing is made by composite action of a number of negatives at different focal distances. Then we will have some astonishing revelations, such as we have synthetically in Marey's or the kinetoscopic photographs of objects in motion. Certainly with the wonderful improvements in high power microscopy and in rapid photography the correct combination will come soon. Meantime, Heitzmann's master drawings are not only better, but better evidence than present photographs.

Heitzmann's answer to doubt is the only correct one,—come and see. Come to his laboratory and learn how to see. Any one who is unable to see the reticulum does not know how to see it. He that will not accept Heitzmann's drawings will not accept even the coming photographs, and probably he that will not accept these evidences is not willing to go to the expense of time and trouble to learn how to see for himself; certainly that individual expense should not be all saddled on Heitzmann or Bödecker, *et al.*

F. A. Roy.

## Figures and Tables

**Figure f1:**